# Antisaccade Performance in Adolescent Obsessive–Compulsive Disorder: Evidence from a Familial-Risk Design

**DOI:** 10.3390/jemr19040078

**Published:** 2026-07-15

**Authors:** Oguz Bilal Karakus, Ayse Akca, Muhammed Said Demirkan, Zeynep Ahsen Ozcan, Didem Cek Ozturk, Ilayda Barankoglu Sevin, Sumeyra Karakus, Ibrahim Selcuk Esin, Onur Burak Dursun

**Affiliations:** 1 Department of Child and Adolescent Psychiatry, Trabzon Kanuni Training and Research Hospital, Trabzon 61290, Turkey; 2 BEH Children and Young People Mental Health Services, North London NHS Foundation Trust, Edgware Community Hospital, Burnt Oak Broadway, Edgware HA8 0AD, UK; 3 Department of Child and Adolescent Psychiatry, Dr. Behcet Uz Training and Research Hospital, Izmir 35210, Turkey; 4 Department of Infectious Diseases and Clinical Microbiology, Trabzon Kanuni Training and Research Hospital, Trabzon 61290, Turkey

**Keywords:** obsessive–compulsive disorder, antisaccade, oculomotor inhibition, endophenotype, unaffected siblings, adolescents

## Abstract

This study aimed to compare antisaccade performance among adolescents with obsessive–compulsive disorder (OCD), their unaffected siblings, and healthy controls, and to examine whether this performance may serve as a candidate familial-risk marker for OCD. The study included 48 adolescents aged 12–18 years with OCD, 35 unaffected siblings, and 39 healthy controls. Participants completed an antisaccade task using an eye-tracking device. Antisaccade correct response rate and latency were evaluated as the primary outcomes, whereas saccadic velocity was examined as an exploratory measure of oculomotor execution. The OCD group showed lower antisaccade correct response rate and longer latency compared with healthy controls, whereas the sibling group demonstrated intermediate performance. Exploratory analyses identified statistically significant group differences in mean saccadic velocity; however, this finding should be interpreted cautiously. Regression analyses indicated that both OCD diagnosis and sibling status were independently associated with antisaccade correct response rate and latency, whereas findings for saccadic velocity should be considered exploratory. These findings suggest that impairments in antisaccade performance may not be specific to OCD but may also be present, albeit to a milder extent, in unaffected siblings, providing preliminary evidence supporting antisaccade performance as a candidate familial-risk marker for OCD that warrants further investigation within an endophenotype framework.

## 1. Introduction

Obsessive–compulsive disorder (OCD) is a chronic psychiatric disorder characterized by marked functional impairment, with a lifetime prevalence of approximately 1–3% [1]. Because of the high disease burden it causes, the World Health Organization lists OCD among the disorders that lead to the greatest global functional disability [2]. The clinical burden of the disorder is not limited to the individual level; it also has serious economic and psychosocial consequences for families and society [3]. However, the often internalized nature of its symptoms and the difficulty patients experience in disclosing them often lead to delays in diagnosis and intervention [4].

Neurobiological models point to functional abnormalities in cortico-striato-thalamo-cortical circuits in the pathophysiology of OCD. In particular, irregular patterns of activity between frontal regions, such as the orbitofrontal cortex and anterior cingulate cortex, and basal ganglia structures, are thought to be associated with disruptions in executive functions such as cognitive control and response inhibition [5,6]. Indeed, it has been suggested that deficits in behavioral and cognitive inhibition may play a central role in the inability to suppress obsessive thoughts and in the continuation of compulsive behaviors [7].

Despite the strong genetic basis of OCD, genome-wide association studies have shown that it is difficult to establish direct and consistent relationships between the clinical phenotype and specific genetic variants [8]. For this reason, the endophenotype approach, which focuses on intermediate markers between genetic risk and clinical symptoms, provides a useful conceptual framework for understanding the etiology of complex psychiatric disorders such as OCD. Endophenotypes are measurable characteristics that are relatively independent of clinical state, heritable, and more pronounced in patients and in first-degree relatives who are unaffected by the disorder than in healthy controls [9]. In OCD, response inhibition and executive control deficits have been proposed as potential candidate endophenotypes because they appear to satisfy several of these criteria [10]. Studies involving unaffected first-degree relatives are particularly valuable for identifying cognitive markers associated with the underlying neurobiology of OCD. Impairments in executive functions such as inhibition, planning, and decision-making observed in these individuals may emerge independently of clinical state, suggesting that these deficits may reflect trait-related rather than state-dependent characteristics [11].

Traditional neuropsychological tests and computerized tasks used to assess response inhibition, such as Go/No-Go and Stop-Signal paradigms, provide millisecond-level reaction time information, but they often cannot fully separate inhibition processes from concurrent cognitive components such as attention, stimulus discrimination, and response selection [12]. For example, performance on the Go/No-Go task involves not only response suppression capacity, but also the accurate discrimination of No-Go stimuli and, in some cases, ignoring these stimuli rather than actively suppressing a response to them [13]. In addition, some stimulus pairings used in the task (for example, red for Go and green for No-Go) may conflict with everyday learned associations and thereby affect performance. Similarly, although the Stop-Signal paradigm appears to provide a “purer” measure of inhibition, inhibitory performance in this task is determined by a calculated value rather than a directly observed behavior. Furthermore, the auditory stop cues used in the task may also influence performance, which can complicate interpretation of the findings [14]. Given these limitations, there is a need for experimental approaches that can assess inhibitory control processes in a more isolated and direct manner. In this context, oculomotor tasks offer important advantages as they allow the suppression of reflexive responses and the initiation of voluntary actions to be examined within a single behavioral paradigm [15]. Among these paradigms, the antisaccade task has become one of the most widely used experimental approaches for investigating inhibitory control in psychiatric research. Accordingly, antisaccade performance has increasingly been conceptualized as a transdiagnostic marker of executive dysfunction across a broad range of psychiatric disorders, extending beyond OCD alone. A recent meta-analysis including multiple psychiatric disorders demonstrated that antisaccade abnormalities constitute a robust transdiagnostic dimension of inhibitory-control dysfunction across psychiatric diagnoses [15]. Furthermore, elevated antisaccade error rates have also been reported in individuals at clinical high risk for psychosis and in unaffected relatives of patients with schizophrenia, supporting the potential utility of antisaccade performance both as an indicator of familial liability and as a candidate marker for endophenotype-oriented research [16]. More recently, eye-tracking paradigms have been increasingly investigated as potential digital biomarkers for detecting subtle neurocognitive alterations across psychiatric disorders, further highlighting their translational potential for early detection and risk stratification [17]. The antisaccade task requires suppression of the automatic gaze shift (prosaccade) that occurs when a peripheral stimulus appears and instead the generation of a voluntary eye movement toward the location directly opposite the stimulus. In this task, measures such as antisaccade error rate and latency provide direct and objective indicators of the inhibition of reflexive responses and the efficiency of voluntary control [18]. With eye-tracking technology, these processes can be recorded with high temporal and spatial resolution; thus, behavioral outputs associated with fronto-striatal networks involved in inhibitory control can be evaluated with precision [15].

Among antisaccade performance measures, antisaccade correct response rate is widely considered a behavioral index of inhibitory control, as successful task performance requires suppression of a reflexive prosaccade and generation of a voluntary saccade in the opposite direction. In contrast, antisaccade latency reflects the efficiency of voluntary response preparation and initiation processes, whereas saccadic velocity provides information regarding oculomotor execution and motor output [15,18]. Previous studies have reported abnormalities across these measures in individuals with OCD, suggesting impairments in inhibitory control and executive functioning [12,19]. Accordingly, these metrics may provide complementary information regarding the neurocognitive mechanisms underlying OCD and may serve as candidate markers of familial vulnerability and potential endophenotypic risk [19,20].

Studies examining antisaccade performance in OCD have generally reported higher error rates and/or prolonged latencies in patients compared with healthy controls; however, these findings have been inconsistent across studies [12,21,22]. Such variability may partially reflect methodological differences among studies, including differences in participant characteristics (e.g., medication status, psychiatric comorbidity, and inclusion/exclusion criteria), eye-tracking systems and sampling frequencies, antisaccade task design (e.g., number and eccentricity of target locations), and data-processing procedures used to quantify oculomotor performance. This heterogeneity suggests that antisaccade performance may reflect multiple underlying processes rather than a single deficit, highlighting the need for a more detailed examination of its components. In this context, evaluating antisaccade performance within an endophenotype framework may provide additional insight into the neurocognitive mechanisms underlying OCD. Nevertheless, studies addressing antisaccade performance as a candidate endophenotype remain limited. Existing evidence, largely derived from adult samples, has reported similar oculomotor abnormalities in individuals with OCD and their unaffected first-degree relatives, supporting the possibility that these alterations may be related to familial risk rather than clinical state alone [19,20].

Antisaccade performance has been shown to undergo marked developmental changes from childhood through adolescence, with error rates decreasing as age increases [23]. However, adolescence represents a developmental stage during which prefrontal and fronto-striatal networks involved in executive control are still maturing [24,25]. In this context, examining antisaccade performance in adolescents may provide a valuable opportunity to detect alterations in inhibitory control before full neurocognitive maturation is achieved. Moreover, because most previous studies investigating antisaccade performance and familial risk in OCD have been conducted in adult populations, evidence from adolescent samples remains relatively limited.

To the best of our knowledge, no previous study has examined antisaccade performance as a candidate marker of familial vulnerability by comparing adolescents with OCD, their unaffected siblings, and healthy controls. The present study therefore aimed to compare antisaccade performance among adolescents with active OCD, their siblings without an OCD diagnosis, and healthy controls with no personal or family history of OCD, and to investigate whether oculomotor inhibitory control measures may represent a candidate marker of familial vulnerability to OCD. By focusing on an adolescent sample and simultaneously examining patients, unaffected siblings, and healthy controls, this study seeks to extend previous adult-oriented findings and provide further insight into the relationship between inhibitory control, familial vulnerability, and OCD. In this framework, we hypothesized that individuals with OCD and their unaffected siblings would exhibit lower antisaccade correct response rate and longer antisaccade latencies compared with healthy controls.

## 2. Materials and Methods

### 2.1. Study Design and Participants

This study was conducted at the Child and Adolescent Psychiatry Clinic of a training and research hospital in Türkiye between 1 October 2024 and 1 October 2025. The study sample consisted of adolescents diagnosed with obsessive–compulsive disorder (OCD; n = 48), unaffected siblings of individuals with OCD (SIBL; n = 35), and healthy controls (HC; n = 39). Prior to the study, all participants were provided with detailed information about the study, and written informed consent was obtained from both the adolescents and their parents.

The OCD group included adolescents aged 12–18 years who met the *Diagnostic and Statistical Manual of Mental Disorders, Fifth Edition, Text Revision* (DSM-5-TR) criteria for OCD, with diagnoses confirmed by a semi-structured clinical interview (Kiddie Schedule for Affective Disorders and Schizophrenia for School-Age Children, K-SADS-5). To ensure the inclusion of clinically active cases, individuals in remission or with subthreshold symptoms were excluded; accordingly, only participants with a total score greater than 14 on the Children’s Yale-Brown Obsessive Compulsive Scale (CY-BOCS) were included [26]. The SIBL group consisted of siblings of individuals with OCD and included adolescents aged 12–18 years who did not meet DSM-5-TR criteria for OCD and had no psychiatric disorder identified during the K-SADS-5 assessment. The HC group was recruited from adolescents attending other outpatient clinics of the same hospital for non-psychiatric medical complaints (e.g., headache, upper respiratory tract infection, or abdominal pain). To minimize the potential influence of acute illness on eye-tracking performance, participants completed the assessment only after the presenting medical condition had clinically resolved or stabilized. None of the healthy control participants was receiving medication at the time of the eye-tracking assessment. These participants had no psychiatric diagnosis based on K-SADS-5 assessment and no history of OCD in first-degree relatives. Among the 35 unaffected siblings, 13 were siblings of OCD participants who were also included in the present study, whereas the remaining 22 siblings were related to individuals with OCD who were not enrolled in the study. All healthy controls were recruited from unrelated families.

Exclusion criteria for all groups included the presence of neurological disorders, particularly epilepsy; any condition significantly affecting vision or uncorrected visual impairment; and the presence of cognitive impairment. In addition, for the OCD group, the presence of any comorbid psychiatric disorder according to K-SADS-5 was considered an exclusion criterion. Furthermore, in the OCD group, the use of any psychotropic medication other than antidepressants prescribed for OCD treatment (e.g., antipsychotics, benzodiazepines, or psychostimulants) was an exclusion criterion.

The sample size was calculated using G*Power software version 3.1.9.7. Based on a previous study [20], with an effect size (f) of 0.44, an alpha level of 0.05, and a statistical power of 95%, the minimum required total sample size was calculated as 105, with at least 35 participants targeted for each group. Accordingly, 125 participants meeting inclusion criteria for the OCD group were initially screened. Of these, 10 were excluded due to comorbid medical conditions (epilepsy [n = 2], cerebral palsy [n = 1], visual impairment [n = 7]). 7 participants were excluded due to the use of antipsychotics (n = 5) or benzodiazepines (n = 2). Among the remaining participants, 57 were excluded because of at least one comorbid psychiatric disorder. Because some participants met criteria for more than one psychiatric diagnosis, the total number of comorbid diagnoses exceeded the number of excluded participants (attention-deficit/hyperactivity disorder [n = 26], major depressive disorder [n = 18], anxiety disorders [n = 27], tic disorder [n = 4], intellectual disability [n = 8], autism spectrum disorder [n = 3], psychotic disorder [n = 2], bipolar disorder [n = 1]). Following these exclusions, 51 participants remained eligible. An additional 3 participants were unable to complete the antisaccade task. Consequently, 48 participants completed the study in the OCD group. For the SIBL group, 48 eligible participants were identified; 13 were excluded based on exclusion criteria, and 35 participants completed the study. For the HC group, 42 participants were invited; 2 withdrew from the study and 1 was unable to complete the antisaccade task, resulting in 39 participants completing the study.

### 2.2. Procedure

The study protocol was approved by the Scientific Research Ethics Committee of University of Health Sciences Türkiye, Trabzon Faculty of Medicine (Approval No: 2025/105; Approval Date: 13 August 2024), and all procedures were conducted in accordance with the Declaration of Helsinki (1975, revised in 2013). Written informed consent was obtained from both participants and their parents/legal guardians. The assessment was carried out in two stages. In the first stage, participants’ sociodemographic and clinical information was recorded, diagnostic evaluation was performed using K-SADS-5 in accordance with DSM-5-TR criteria, and OCD symptom severity was assessed using the CY-BOCS. Participants who met the study criteria were then scheduled for a second stage, during which the antisaccade task was administered and eye-tracking data were recorded simultaneously. Prior to the second stage, participants were instructed to obtain adequate sleep, avoid fasting, and refrain from consuming stimulants such as caffeine.

The second stage was conducted in a dedicated assessment room with stable ambient lighting and minimal background noise. Participants were tested individually, unnecessary visual and auditory distractions were minimized, and the same environmental conditions were maintained across all assessments. Before the eye-tracking task, task instructions were explained and a brief practice session consisting of five trials was administered. Following successful completion of the practice session, each participant completed 48 antisaccade trials. The total duration of the second stage (including calibration and practice) was approximately 25–30 min per participant.

No blinding procedures were implemented during task administration, eye-tracking data preprocessing, or statistical analyses. Nevertheless, eye-tracking data preprocessing was performed according to predefined objective criteria established before data analysis and applied uniformly to all participants.

### 2.3. Measurement Tools

#### 2.3.1. Sociodemographic Data Form

Sociodemographic and clinical characteristics of the participants were collected using a sociodemographic data form developed by the researchers. This form included information on age, sex, educational status, monthly income, psychiatric history, duration of illness, and treatments used, and was completed by the interviewer during face-to-face interviews.

#### 2.3.2. Kiddie Schedule for Affective Disorders and Schizophrenia for School-Age Children (K-SADS-5)

This semi-structured interview, developed by Kaufman et al., is designed to assess past and current psychopathology in children and adolescents according to DSM diagnostic criteria [27]. The validity and reliability of the Turkish version of the scale has been established [28]. In the present study, OCD and other psychiatric disorders were assessed using this interview.

#### 2.3.3. Children’s Yale-Brown Obsessive Compulsive Scale (CY-BOCS)

Developed by Scahill et al., the Children’s Yale-Brown Obsessive Compulsive Scale (CY-BOCS) is a clinician-administered semi-structured instrument used to assess the severity of obsessive-compulsive symptoms in children and adolescents [29]. The scale consists of 10 clinician-rated items evaluating the severity of obsessions and compulsions across dimensions including time occupied, interference, distress, resistance, and degree of control. Five items assess obsessions and five assess compulsions, yielding Obsession and Compulsion subscale scores ranging from 0 to 20 and a total score ranging from 0 to 40. Higher scores indicate greater OCD symptom severity. The validity and reliability of the Turkish version of the scale have been established [30]. In the present study, CY-BOCS Obsession, Compulsion, and Total scores were used to quantify OCD symptom severity and to examine their associations with antisaccade performance measures.

### 2.4. Eye-Tracking System

Eye movement data were recorded using a Gazepoint GP3 HD^®^ eye tracker (Gazepoint Research Inc., Vancouver, BC, Canada) with a sampling frequency of 250 Hz. The antisaccade task was created using PsychoPy v2025.2.4 software. Stimuli were presented on a 17-inch monitor with a resolution of 1920 × 1080 pixels, and participants were tested in a seated position at a viewing distance of 41 cm [20]. To minimize artifacts related to head and body movements during measurement, an adjustable chin rest was used.

Before eye-tracking assessment, ocular dominance was assessed using the Dolman method (hole-in-the-card test), and eye movement data were recorded from the dominant eye to reduce potential confounding effects of dominance differences on measurements [31]. A standard 9-point calibration procedure was performed prior to data acquisition. Calibration quality was subsequently verified using the built-in validation procedure by confirming that the recorded gaze position corresponded accurately to each calibration point. Recording was initiated only after satisfactory calibration accuracy had been achieved. Whenever calibration accuracy was considered unsatisfactory, the calibration and validation procedures were repeated until acceptable calibration quality was obtained.

### 2.5. Antisaccade Task and Performance Measures

The antisaccade task began with a white square fixation stimulus subtending approximately 2° × 2° of visual angle, presented on a black background. This stimulus remained on the screen for a randomly varying duration between 800 and 1200 ms to prevent anticipatory effects that could influence performance. Two hundred milliseconds after the fixation stimulus disappeared, a white circular target stimulus (0.3 cm in diameter) appeared on the screen for 800 ms. The target was presented at one of four possible locations corresponding to ±6° and ±12° of visual angle from the center. Target location was pseudorandomized across trials, ensuring an equal number of presentations at each eccentricity and direction. Subsequently, a black screen was displayed for a randomly varying duration of 500–1000 ms before the next antisaccade trial began [32].

Participants were instructed to shift their gaze to the mirror-opposite location of the target without looking directly at the target itself. Consistent with previous antisaccade studies [22,32], saccades occurring within the first 80 ms following target onset or after 600 ms were excluded from analysis because they are likely to represent anticipatory responses or delayed responses associated with attentional lapses [33]. Additionally, trials were excluded if, despite an initially correct saccade direction, the eye crossed the midline (approximately ±0.8° of visual angle) and moved toward or fixated on the target during the trial, or if they contained recording artifacts, including blink-related signal loss, looking off-screen, or corrupted corneal reflection signals. These artifacts were identified during offline review and confirmed by the absence of reliable gaze-coordinate data in the exported eye-tracking recordings. These preprocessing criteria were adopted to improve the reliability of antisaccade performance measures by minimizing the influence of recording artifacts and responses that did not adequately reflect task performance [22]. Raw gaze-coordinate data recorded with the Gazepoint GP3 HD^®^ eye tracker were exported for offline analysis and processed according to predefined preprocessing criteria before area-of-interest (AOI)-based classification of antisaccade responses. For each trial, a predefined AOI corresponding approximately to the size of the target stimulus was defined at the mirror-symmetric antitarget location (e.g., −6° for a +6° target and +12° for a −12° target). A response was classified as a correct antisaccade only when the first valid gaze shift entered the corresponding antitarget AOI. Thus, for targets presented at +6° or +12°, only gaze shifts entering the corresponding −6° or −12° antitarget AOI were classified as correct, and vice versa. Responses directed merely to the opposite visual hemifield were not classified as correct antisaccades. Likewise, because separate AOIs were defined for the ±6° and ±12° antitarget locations, gaze shifts landing between these predefined antitarget locations did not enter either AOI and were therefore not classified as correct antisaccades.

Antisaccade performance was evaluated using three complementary outcome measures. *Antisaccade correct response rate* and *mean correct antisaccade latency* were considered the principal outcome measures because they are generally regarded as the primary indicators of inhibitory control and the efficiency of voluntary response generation. The *antisaccade correct response rate (%)* was calculated as the proportion of valid correct antisaccades relative to the total number of valid antisaccade trials. For each valid correct antisaccade, latency (ms) was defined as the time interval between target onset and the initiation of the first valid saccade directed toward the corresponding antitarget AOI. *Mean correct antisaccade latency* for each participant was calculated by averaging latency values across all valid correct antisaccades. For each valid correct antisaccade, saccadic velocity (°/ms) was calculated offline as the ratio of saccade amplitude to saccade duration. Saccade amplitude was defined as the angular displacement between the initial and final gaze positions of the first valid saccade directed toward the corresponding antitarget AOI. Velocity values are reported throughout the manuscript in degrees per millisecond (°/ms); for ease of interpretation, the observed values correspond approximately to 260–300°/s. *Mean correct antisaccade velocity* for each participant was calculated by averaging velocity values across all valid correct antisaccades. This measure was included as a complementary exploratory outcome to provide additional information regarding oculomotor execution. Although velocity may contribute to the characterization of oculomotor performance, its relationship with inhibitory-control mechanisms and familial vulnerability remains less consistently established than that of response accuracy and latency.

For each participant, trial-level eye-tracking data were aggregated to calculate the antisaccade correct response rate, mean correct antisaccade latency, and mean correct antisaccade velocity. These participant-specific measures were subsequently used in all group comparisons and regression analyses.

**Figure 1:**
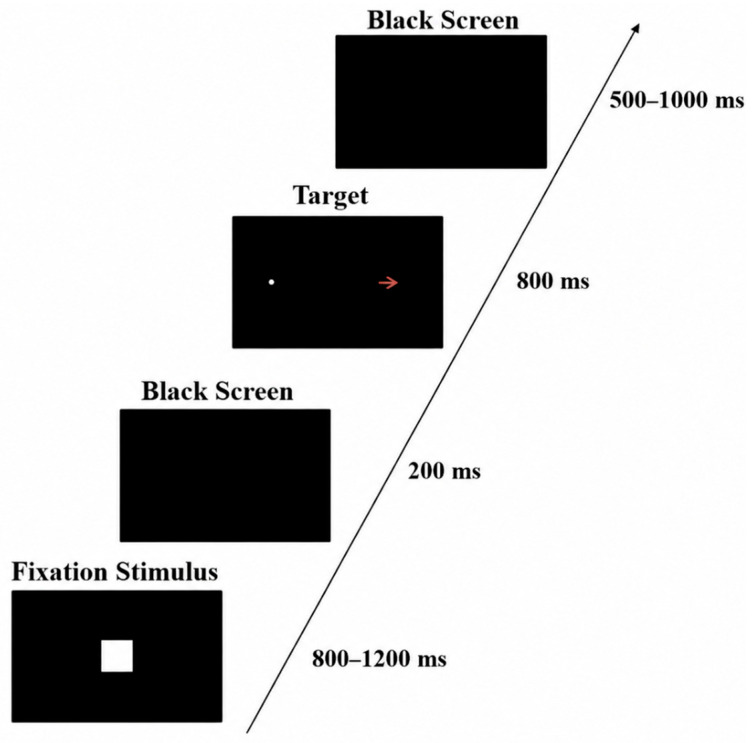
Experimental Design of the Antisaccade Task. The figure is not drawn to scale and does not represent the actual spatial arrangement of stimuli. Target stimuli were presented along the horizontal meridian at eccentricities of ±6° and ±12° of visual angle.

A schematic representation of the experimental paradigm is presented in Figure 1.

### 2.6. Statistical Analysis

Data were examined for missing values and outliers. No missing data were identified in the dataset. No outliers were removed from the analyses because all identified values were considered plausible observations and did not reflect data-entry errors or measurement artifacts. The distribution of continuous variables was assessed using the Kolmogorov–Smirnov test and visual inspection of histograms. The chi-square test was used to compare categorical variables between groups. For comparisons of continuous variables across groups, one-way ANOVA was used when the normality assumption was met, whereas the Kruskal–Wallis H test was used when it was not. In ANOVA analyses, Bonferroni correction was used as the post hoc test; for the Kruskal–Wallis H test, Mann–Whitney U tests with Bonferroni correction were performed. Associations between continuous variables were examined using Pearson correlation analysis when the normality assumption was satisfied and Spearman rank-order correlation analysis when it was not. In addition to the statistical analyses, effect sizes were calculated and reported where appropriate for group comparisons (for ANOVA: partial η^2^; for Kruskal–Wallis: ε^2^; for Mann–Whitney U: rank-biserial correlation [r]; for chi-square: Cramer’s V).

Hierarchical multiple linear regression analyses were performed to examine factors predicting antisaccade performance. In the analyses, antisaccade correct response rate was entered as the dependent variable, and age and sex, along with dummy-coded variables representing group membership (OCD and SIBL; reference group: HC), were entered into the model sequentially as independent variables.

In addition, similar regression models were constructed for mean correct antisaccade latency and antisaccade velocity. Because these variables did not meet the assumption of normality in the initial analyses, logarithmic transformation was applied before regression modeling. Following transformation, normality was reassessed using the same procedures (Kolmogorov–Smirnov tests and visual inspection of histograms) before inclusion in the regression models. In all regression analyses, model assumptions (linearity, multicollinearity, normality of residuals, and homoscedasticity) were checked. As a sensitivity analysis, all regression models were repeated using family-clustered robust standard errors, with family ID specified as the clustering variable, to account for the potential non-independence of observations arising from related OCD–sibling pairs.

All analyses were conducted using the Statistical Package for the Social Sciences (SPSS), version 27.0. The level of statistical significance was set at α = 0.05.

## 3. Results

**Figure 2:**
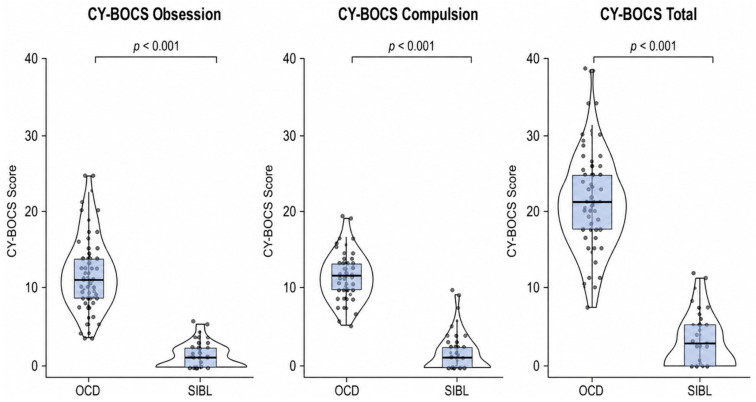
Distribution of CY-BOCS Obsession, Compulsion, and Total Scores in Adolescents with OCD and SIBL Groups. Group comparisons were conducted using independent-samples *t* tests. Participants with OCD showed significantly higher Obsession, Compulsion, and Total CY-BOCS scores than unaffected siblings (all *p* < 0.001).

As shown in Table 1, no significant differences were found between the groups in terms of age, sex, or family income level (all *p* values > 0.05). When the OCD and SIBL groups were compared in terms of CY-BOCS scores, participants in the OCD group had higher Obsession, Compulsion, and Total scores (all *p* values < 0.001). Figure 2 visually demonstrates these differences, showing markedly higher CY-BOCS Obsession, Compulsion, and Total scores in the OCD group compared with the SIBL group.

Before examining antisaccade performance, trial-retention rates were evaluated to determine whether preprocessing-related data loss differed across groups. The proportion of excluded antisaccade trials was low in all groups. Given that each participant completed 48 antisaccade trials, the mean number of excluded trials was 4.23 ± 3.04 in the OCD group, 3.77 ± 2.80 in the SIBL group, and 3.15 ± 2.82 in the HC group, corresponding to trial-retention rates of 91.2%, 92.1%, and 93.4%, respectively. A one-way ANOVA revealed no significant group difference in the number of excluded trials (*F*_(2119)_ = 1.479, *p* = 0.232), indicating comparable data quality and trial-retention rates across groups. Because latency and velocity were calculated only from valid correct antisaccades, we additionally examined the mean number of valid correct antisaccade trials contributing to these estimates. This number differed significantly among the groups (*F*_(2119)_ = 23.99, *p* < 0.001), with OCD participants contributing fewer valid correct trials (18.8 ± 8.9) than SIBL (24.1 ± 5.9) and HC (30.6 ± 8.3).

**Figure 3:**
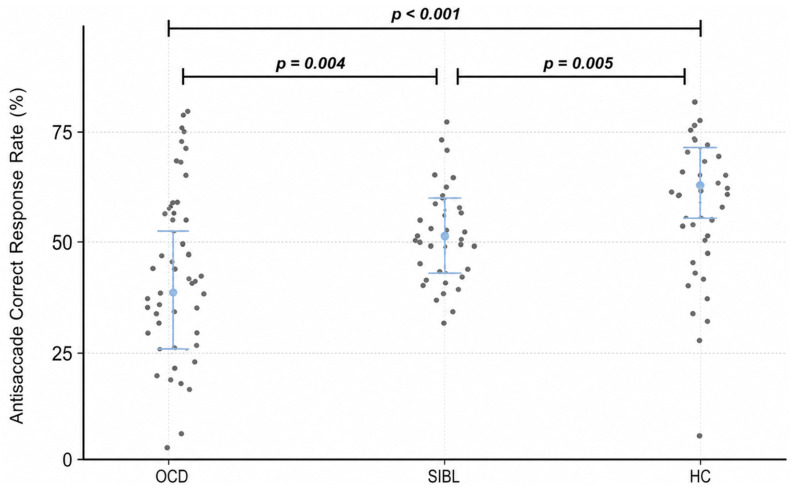
Comparison of Antisaccade Correct Response Rate Across Groups. Error bars represent 95% confidence intervals. A one-way ANOVA showed a significant group difference, *F*_(2119)_ = 23.506, *p* < 0.001. Post hoc comparisons indicated significant differences between OCD and SIBL (*p* = 0.004), OCD and HC (*p* < 0.001), and SIBL and HC (*p* = 0.005).

**Figure 4:**
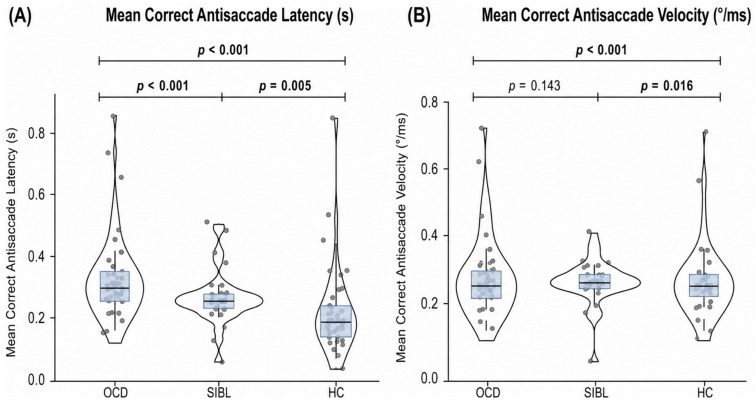
Comparison of Mean Correct Antisaccade Latency and Velocity Across Groups. (**A**) Mean correct antisaccade latency and (**B**) mean correct antisaccade velocity across OCD, SIBL, and HC groups. Violin plots illustrate score distributions, boxes represent the interquartile range (IQR), horizontal lines indicate medians, and gray dots represent individual participants. Group differences were evaluated using the Kruskal–Wallis test followed by Bonferroni-corrected Mann–Whitney U post hoc comparisons. Pairwise comparison results are displayed above the plots.

Results regarding antisaccade performance measures are presented in Table 2 and Figure 3 and Figure 4.

A significant difference was found among the groups in antisaccade correct response rate (*F*_(2119)_ = 23.506, *p* < 0.001, *Partial η*^2^ = 0.283). Post hoc analyses showed that the OCD group had a lower antisaccade correct response rate than both the SIBL group (*p* = 0.004) and the HC group (*p* < 0.001). In addition, the SIBL group had a lower antisaccade correct response rate than the HC group (*p* = 0.005).

A significant group difference was also observed in mean correct antisaccade latency (*χ*^2^_(2)_ = 26.900, *p* < 0.001, *ε*^2^ = 0.209). Post hoc analyses showed that latency was higher in the OCD group than in both the SIBL group (*p* < 0.001) and the HC group (*p* < 0.001), and higher in the SIBL group than in the HC group (*p* = 0.005).

Exploratory analyses identified a statistically significant group difference in mean correct antisaccade velocity (*χ*^2^_(2)_ = 12.878, *p* = 0.002, *ε*^2^ = 0.091). Post hoc analyses indicated that velocity was lower in both the OCD group (*p* < 0.001) and the SIBL group (*p* = 0.016) than in the HC group; however, no significant difference was observed between the OCD and SIBL groups (*p* = 0.143). Despite the statistically significant overall group difference, considerable overlap in individual velocity values was observed across groups (Figure 4B).

As a sensitivity analysis, additional ANCOVA models were performed with age included as a covariate (Supplementary Table S1). The age-adjusted analyses generally supported the primary findings. Group differences in antisaccade correct response rate remained statistically significant after age adjustment (*F*_(2116)_ = 19.610, *p* < 0.001). For mean correct antisaccade latency, the overall group effect also remained statistically significant; however, after age adjustment, the pairwise differences between the OCD and SIBL groups and between the SIBL and HC groups were attenuated and no longer reached statistical significance. For mean correct antisaccade velocity, the overall pattern of group differences was similar; however, the overall group effect no longer reached statistical significance after age adjustment. The values presented in Supplementary Table S1 represent age-adjusted estimated marginal means derived from the ANCOVA models, whereas the values reported in Table 2 represent the observed group means. Therefore, small differences between the two tables are expected and reflect the statistical adjustment for age.

Among participants in the OCD group, 21 were receiving no medication, whereas 27 were receiving antidepressant treatment for OCD. Among the medicated participants with OCD, 13 were taking fluoxetine, 9 sertraline, 2 escitalopram, 2 fluvoxamine, 3 clomipramine, 2 mirtazapine, 1 trazodone, and 1 agomelatine. No significant differences were found between medicated and unmedicated participants in the OCD group with respect to antisaccade correct response rate, latency, or velocity (all *p* values > 0.05; Supplementary Table S2).

Data on the associations between antisaccade performance and clinical and demographic characteristics of the participants are presented in Table 3.

Accordingly, when all participants were considered, age showed a significant positive association with antisaccade correct response rate (*r* = 0.268, *p* = 0.003) and a significant negative association with mean correct antisaccade latency (*r* = −0.187, *p* = 0.040). No significant association was found between age and mean correct antisaccade velocity (*p* > 0.05). No significant association was observed between sex and antisaccade performance (all *p* values > 0.05). In analyses specific to the OCD group, no significant association was found between CY-BOCS scores and antisaccade performance measures (all *p* values > 0.05). Similarly, no significant association was found between illness duration and antisaccade performance (all *p* values > 0.05).

Hierarchical regression analysis was conducted to examine factors predicting antisaccade correct response rate (Table 4). In the first model, age and sex were included, and the model was significant (*R*^2^ = 0.080, Adjusted *R*^2^ = 0.065, *p* = 0.007); in this model, only age was a significant predictor (*p* = 0.004). With the addition of the variable representing the OCD group (OCD dummy), the explanatory power of the model increased significantly (Δ*R*^2^ = 0.185, *p* < 0.001), and being in the OCD group was found to be negatively associated with antisaccade correct response rate (*β* = −0.436, *p* < 0.001). With the addition of the SIBL dummy variable in the final model, the explanatory power of the model increased further (Δ*R*^2^ = 0.052, *p* = 0.004), and the final model explained 31.7% of the total variance (*R*^2^ = 0.317, Adjusted *R*^2^ = 0.293). In the final model, belonging to either the OCD group (*β* = −0.576, *p* < 0.001) or the SIBL group (*β* = −0.266, *p* = 0.004) was associated with a lower antisaccade correct response rate compared with healthy controls. In the models predicting mean correct antisaccade latency and velocity (Supplementary Tables S3 and S4), analyses after logarithmic transformation showed that the transformed variables no longer demonstrated substantial deviations from normality based on Kolmogorov–Smirnov tests and visual inspection of histograms. Belonging to the OCD and SIBL groups was associated with longer latency periods (*β* = 0.448, *p* < 0.001; *β* = 0.209, *p* = 0.037, respectively). Similarly, both groups were associated with lower antisaccade velocity (OCD: *β* = −0.223, *p* = 0.037; SIBL: *β* = −0.215, *p* = 0.041), although the explanatory power of this model was limited (*R*^2^ = 0.075). Inspection of the variance inflation factor (VIF) values indicated no evidence of problematic multicollinearity among the predictor variables. Sensitivity analyses using family-clustered robust standard errors demonstrated that the overall pattern of findings remained unchanged. After accounting for family clustering, OCD diagnosis and sibling status remained significantly associated with antisaccade correct response rate, antisaccade latency, and exploratory measure of saccadic velocity (Supplementary Table S5).

## 4. Discussion

This study compared antisaccade performance among adolescents with OCD, their unaffected siblings, and healthy controls to examine the relationship between oculomotor inhibitory control processes and both OCD and familial vulnerability. The findings showed significant group differences across the primary antisaccade performance measures, namely antisaccade correct response rate and latency, while exploratory analyses additionally identified group differences in saccadic velocity. The overall pattern of performance, being lowest in the OCD group, intermediate in the sibling group, and highest in healthy controls, suggests that inhibitory control processes may reflect not only an impairment associated with the disorder but also a pattern that may be related to familial vulnerability.

One of the important contributions of this study is that antisaccade performance was evaluated in an adolescent sample within a familial-risk design, together with its correct response rate, latency, and velocity components. Whereas antisaccade correct response rate is generally considered to reflect the ability to suppress a reflexive saccadic response, latency and velocity provide information about different components of voluntarily generating a saccade in the opposite direction [34]. Latency is associated with response initiation and cognitive control processes [35,36], whereas saccadic velocity is thought to reflect processes more closely related to motor execution and arousal level [37]. Examining these parameters together allows a more detailed assessment of the processes underlying antisaccade performance.

The findings regarding antisaccade correct response rate indicate a marked inhibitory deficit in the OCD group. Although increased antisaccade error rates in OCD have been frequently reported in the literature, it is known that findings may vary depending on task characteristics and sample differences [22]. Overall, multiple studies have consistently shown increased antisaccade error rates in both individuals with OCD and their unaffected first-degree relatives [12,20,38,39]. Similarly, studies evaluating executive functions with methods other than antisaccade tasks have also reported difficulties in inhibitory control in both OCD groups and relatives [40,41,42]. In the present study, the reduced performance observed in the sibling group compared with controls, supported by the regression analyses, suggests that differences in inhibitory control processes may not be solely secondary to clinical status and may instead include a measurable familial vulnerability component.

The findings for antisaccade latency largely paralleled those for antisaccade correct response rate. The longer latency observed in the OCD group suggests a slowing not only in suppressing the reflexive response but also in initiating the voluntary response. Antisaccade latency is associated with higher-order executive functions involving response planning and initiation. Prolonged antisaccade latency in OCD has previously been reported and has been linked to inefficiency in voluntary response generation [22,43]. There are also studies reporting prolonged latency in unaffected relatives [38]. The intermediate latency values observed in the SIBL group suggest that this cognitive slowing cannot be explained solely by the presence of diagnosis and may instead reflect a broader neurocognitive vulnerability. Although the overall antisaccade latency values observed in the present study were shorter than those reported in some adult OCD studies [20], they were broadly comparable to findings from pediatric OCD studies employing similar antisaccade paradigms [22,32]. Therefore, differences in participant age and developmental stage may contribute to variability in antisaccade latency findings across studies. Similarly, the regression analyses showed that belonging to either the OCD or SIBL group was associated with longer antisaccade latency. However, the age-adjusted analyses suggested a more nuanced pattern. Although the overall group effect for antisaccade latency remained statistically significant, the pairwise differences between the OCD and SIBL groups and between the SIBL and HC groups were attenuated and no longer reached statistical significance after age adjustment. Therefore, compared with antisaccade correct response rate, the latency findings should be interpreted with somewhat greater caution. Taken together, these findings are consistent with meta-analytic evidence suggesting that executive dysfunction in OCD may be considered not only as a categorical feature but also as part of a broader spectrum [44].

As a complementary exploratory finding, reduced mean saccadic velocity was observed in both the OCD and SIBL groups compared with healthy controls. Most previous antisaccade studies have focused on error rate and latency, whereas saccadic velocity has been investigated much less frequently. Here, saccadic velocity showed a pattern that was partially dissociated from the other antisaccade measures. The OCD and SIBL groups showed comparable saccadic velocity values. Both groups also demonstrated lower velocities than healthy controls. This pattern suggests that saccadic velocity may partly reflect processes more closely related to oculomotor execution, motor output, and possibly arousal than to inhibitory control itself. These findings support the view that antisaccade performance cannot be explained by a single cognitive process but instead reflects a multicomponent construct involving cognitive control, response selection, and motor execution. Indeed, recent studies have shown that oculomotor performance in OCD is associated not only with inhibition errors but also with a broader control disturbance involving components such as motor output and task-specific performance efficiency [39]. Our findings regarding saccadic velocity differ from those of Khayrullina et al. [45], who similarly reported group differences in antisaccade error rate and latency but found no significant between-group differences in mean gaze velocity. This discrepancy may reflect methodological differences between the studies, including differences in participant age (adolescent versus adult samples), task characteristics (a neutral antisaccade paradigm in the present study versus an emotional antisaccade paradigm incorporating emotional face stimuli), eye-tracking hardware, and the procedures used to derive velocity measures. In this context, the similar reduction in velocity observed in both the OCD and SIBL groups may indicate the possibility of shared underlying mechanisms. Furthermore, because latency and particularly velocity were calculated only from valid correct antisaccades, participants with OCD contributed fewer valid correct trials than the other groups. Consequently, these estimates may have been derived from a smaller and potentially more selected subset of responses. However, given the limited explanatory power of the regression model and the relatively limited literature on this parameter, this finding should be considered exploratory. Moreover, although statistically significant group differences were observed, substantial overlap in individual velocity distributions indicates that this measure showed limited discriminatory ability and should therefore be interpreted cautiously.

The relationships between age and antisaccade performance are important for interpreting the findings. The increase in antisaccade correct response rate and the decrease in latency with age indicate that inhibitory control processes mature developmentally. This finding is consistent with studies showing that antisaccade performance is age-sensitive [32,46,47]. This demonstrates that antisaccade performance has a strong developmental component and that age should be controlled for, particularly in pediatric samples [48]. In the present study, the selection of a sibling group within a similar age range contributed to a more reliable evaluation of familial effects.

The absence of a significant association between symptom severity and illness duration and antisaccade performance in the OCD group suggests that these performance differences may be independent of symptom level. Similar findings have been reported previously, and antisaccade performance has been suggested to be independent of clinical severity [20,49]. Together, these findings are consistent with the possibility that antisaccade performance may reflect trait-like characteristics rather than being solely associated with current symptom severity. Taken together, the present findings provide preliminary evidence that alterations in antisaccade performance may be associated with familial vulnerability to OCD. However, they should be interpreted as supporting further investigation of antisaccade measures as candidate familial-risk markers rather than establishing antisaccade performance as an endophenotype in adolescent OCD. No significant differences were observed between medicated and unmedicated participants with OCD in terms of antisaccade performance. However, because the medication analyses were based on relatively small and clinically heterogeneous subgroups receiving different antidepressant agents, the absence of statistically significant differences should not be interpreted as evidence that antidepressant treatment has no influence on antisaccade performance. Rather, these findings should be considered preliminary, and larger studies specifically designed to evaluate medication-related effects are needed before firm conclusions can be drawn.

Several limitations should be considered when interpreting the findings of this study. The study was conducted at a single center with a relatively modest sample size, which may limit the generalizability of the findings and reduce the statistical power of subgroup analyses. This limitation is particularly relevant to the comparison between medicated and unmedicated participants with OCD, which may have been underpowered to detect modest medication-related effects on antisaccade performance. The cross-sectional design also prevents evaluation of causal relationships and does not allow the present findings to address essential endophenotype criteria such as heritability, temporal stability, or co-segregation within families. Therefore, although the observed antisaccade alterations in unaffected siblings are compatible with familial vulnerability, they should be interpreted as preliminary evidence that antisaccade performance may represent a candidate endophenotypic marker rather than evidence of an established endophenotype. The antisaccade task used in the study consisted of a total of 48 trials. The relatively limited number of trials may have reduced measurement reliability by preventing sufficient balancing of within-subject variability and by increasing the influence of individual trials on the measurements. However, because the study was conducted in an adolescent sample, a shorter protocol was preferred in consideration of attention span and task compliance. This reflects a methodological trade-off, and it may be useful for future studies to evaluate longer task designs in different samples. Furthermore, latency and saccadic velocity were calculated only from valid correct antisaccades. Because participants with OCD contributed fewer valid correct trials than the sibling and healthy control groups, these estimates were based on fewer observations in the OCD group, which may have increased measurement variability and introduced potential selection bias. Therefore, findings related to latency and, in particular, the exploratory saccadic velocity findings should be interpreted with appropriate caution, as they may reflect a smaller and potentially more selected subset of responses in the OCD group. In addition, the 250 Hz sampling frequency and measurement sensitivity of the eye-tracking system may have limited the precision of temporal measures, particularly saccadic velocity. Quantitative endpoint coordinates were also not retained for subsequent analyses; therefore, horizontal and vertical landing-position deviations and spatial landing accuracy could not be examined directly. Although correct antisaccades were classified using predefined mirror-symmetric antitarget locations during preprocessing, future studies should retain endpoint-level data and report landing-position measures to provide a more comprehensive evaluation of task compliance and oculomotor performance. Future studies should also retain trial-level information regarding stimulus laterality (left- vs. right-sided target presentation) to examine potential differences in antisaccade performance between left- and right-oriented stimuli, particularly in light of recent evidence highlighting functional asymmetries between the cerebral hemispheres [50]. In addition, the original PsychoPy task file is no longer available, and no custom analysis scripts were generated because the analyses were performed using predefined preprocessing procedures and standard statistical software rather than bespoke computational pipelines. Although the experimental paradigm and preprocessing workflow have been described in detail to facilitate reproducibility, the unavailability of the original task file may limit exact replication of the experimental implementation. Participants with psychiatric comorbidities were excluded to reduce potential confounding effects and increase internal validity. Although this approach allowed a more specific examination of antisaccade performance in relation to OCD, it may limit the generalizability of the findings because OCD in adolescence commonly co-occurs with disorders such as anxiety disorders, depression, and ADHD. Therefore, the present findings may be most applicable to a relatively selective subgroup of adolescents with OCD. Future studies including larger and more clinically representative samples, as well as participants with common psychiatric comorbidities, may help clarify the influence of psychiatric comorbidity on antisaccade performance. The SIBL group in this study consisted of siblings without a current psychiatric diagnosis. Although this approach reduced potential confounding effects and allowed familial vulnerability to be examined in a more selective manner, it may not fully reflect the broader population of relatives of individuals with OCD, among whom subclinical obsessive–compulsive, anxiety, and depressive symptoms are relatively common. Consequently, the findings may underestimate the magnitude of familial vulnerability observed in routine clinical settings. In addition, possible differential effects of symptom subtypes in the OCD group, such as contamination/cleaning, religious, aggressive, or multiple symptom patterns, on antisaccade performance were not examined. Given the heterogeneous nature of OCD, separating the effects of different symptom dimensions on cognitive and oculomotor processes represents an important area for future research.

## 5. Conclusions

The present study demonstrated that adolescents with OCD show impairments in the primary antisaccade performance measures, reflected by reduced antisaccade correct response rate and prolonged latency. Exploratory analyses additionally suggested lower mean saccadic velocity in both adolescents with OCD and their unaffected siblings. A similar but less pronounced pattern in the primary antisaccade measures was observed in unaffected siblings, suggesting that alterations in oculomotor inhibitory control may be associated with familial vulnerability to OCD. Nevertheless, the cross-sectional nature of the study precludes conclusions regarding heritability, temporal stability, or endophenotypic status. Therefore, the current findings should be regarded as preliminary evidence supporting the potential relevance of antisaccade performance as a candidate endophenotypic marker associated with familial vulnerability to OCD. Future studies employing larger samples, longitudinal designs, family-based genetic approaches, and more comprehensive antisaccade paradigms will be important for clarifying the clinical and neurobiological significance of these findings.

## Figures and Tables

**Table 1:** Comparison of Demographic and Clinical Characteristics Across Groups.

Variables		OCD (*n* = 48)	SIBL (*n* = 35)	HC (*n* = 39)	Statistics	*p*	Effect Size
Age (years)		15.07 ± 1.52	15.25 ± 2.06	15.84 ± 1.88	*F*_(2119)_ = 2.140	0.122	*Partial η*^2^ = 0.035
Gender (f:m)		26:22	15:20	17:22	*χ*^2^_(2)_ = 1.397	0.497	*Cramer’s V* = 0.107
Family Income Level	Low	15 (31.3)	7 (20.0)	11 (28.2)	*χ*^2^_(4)_ = 3.898	0.420	*Cramer’s V* = 0.179
	Moderate	13 (27.1)	16 (45.7)	16 (41.0)			
	High	20 (41.7)	12 (34.3)	12 (30.8)			
Family Structure (Nuclear Family)		38 (79.2)	31 (88.6)	29 (74.4)	*χ*^2^_(2)_ = 2.425	0.297	*Cramer’s V* = 0.141
CY-BOCS	Obsession	12.15 ± 3.61	1.80 ± 1.95	-	*t*_(75.545)_ = *16.799*	**<0.001**	*Cohen’s d* = 3.424
	Compulsion	12.10 ± 3.00	1.83 ± 2.16	-	*t*_(81)_ = *17.225*	**<0.001**	*Cohen’s d* = 3.829
	Total	23.46 ± 6.36	3.63 ± 3.77	-	*t*_(78.111)_ = *17.755*	**<0.001**	*Cohen’s d* = 3.657

Note: Data are presented as mean ± standard deviation for continuous variables and n (%) for categorical variables. F: one-way ANOVA; χ^2^: chi-square test; t: independent samples *t*-test.

**Table 2:** Comparison of Antisaccade Performance Measures Across Groups.

Antisaccade Performance Measures	OCD (*n* = 48)	SIBL (*n* = 35)	HC (*n* = 39)	Statistics	*p*	Effect Size	Post Hoc
Antisaccade Correct Response Rate (%)	42.09 ± 19.60	54.47 ± 12.66	67.38 ± 17.34	*F*_(2119)_ = 23.506	**<0.001**	*Partial η*^2^ = 0.283	*Bonferroni:* OCD-SIBL (*p* = 0.004) OCD-HC (*p* < 0.001) SIBL-HC (*p* = 0.005)
Mean Correct Antisaccade Latency (s)	0.327 (0.299–0.373)	0.295 (0.268–0.309)	0.247 (0.193–0.319)	*χ*^2^_(2)_ = 26.900	**<0.001**	ε^2^ = 0.209	*Mann–Whitney U (Bonferroni adjusted):* OCD-HC (*p* < 0.001) OCD-SIBL (*p* < 0.001) SIBL-HC (*p* = 0.005)
Mean Correct Antisaccade Velocity (°/ms)	0.262 (0.223–0.295)	0.275 (0.265–0.297)	0.295 (0.274–0.317)	*χ*^2^_(2)_ = *12.878*	**0** **.002**	ε^2^ = 0.091	*Mann Whitney U (Bonferroni adjusted):* OCD-HC (*p* < 0.001) SIBL-HC (*p* = 0.016) OCD-SIBL (*p* = 0.143)

Note: Data are presented as mean ± standard deviation for normally distributed variables and median (interquartile range, Q1–Q3) for non-normally distributed variables. F: one-way ANOVA; χ^2^: Kruskal–Wallis H Test.

**Table 3:** Associations Between Antisaccade Performance and Clinical and Demographic Variables.

Variables		Antisaccade Correct Response Rate (%) ^a^	Mean Correct Antisaccade Latency (s) ^b^	Mean Correct Antisaccade Velocity (°/ms) ^b^
Age (year)		***r* = 0.268, *p* = 0.003**	***r* = −0.187, *p* = 0.040**	*r* = 0.166, *p* = 0.068
Sex *^,c^		*r* = 0.115, *p* = 0.209	*r =* −0.062, *p* = 0.496	*r* = 0.124, *p* = 0.173
CY-BOCS **	Obsession	*r* = 0.061, *p* = 0.680	*r* = 0.240, *p* = 0.101	*r* = −0.115, *p* = 0.437
	Compulsion	*r =* −0.005, *p* = 0.976	*r* = 0.163, *p* = 0.270	*r* = 0.016, *p* = 0.912
	Total	*r =* −0.150, *p* = 0.310	*r* = 0.076, *p* = 0.608	*r* = −0.116, *p* = 0.431
Disease duration (months) **		*r* = 0.167, *p* = 0.257	*r* = 0.095, *p* = 0.519	*r* = −0.147, *p* = 0.317

* Sex was coded as female = 0 and male = 1. ** Calculated only in the OCD group (n = 48). ^a^ Pearson correlation analysis; ^b^ Spearman correlation analysis; ^c^ point-biserial correlation.

**Table 4:** Factors Predicting Antisaccade Correct Response Rate: Hierarchical Regression Analysis.

Variables	*B*	*95% CI for B*	*SE*	*β*	*t*	*p*	*VIF*
**Model I**							
Age	2.917	0.957–4.876	0.990	0.260	2.948	**0** **.004**	1.007
Sex (male, ref: female)	3.712	−3.278–10.701	3.530	0.093	1.051	0.295	1.007
**Model II**							
Age	2.298	0.525–4.071	0.895	0.205	2.566	**0** **.012**	1.024
Sex (male, ref: female)	2.032	−4.272–8.336	3.183	0.051	0.638	0.525	1.017
OCD (dummy)	−17.839	−24.319–−11.359	3.272	−0.436	−5.451	**<0.001**	1.028
**Model III**							
Age	1.958	0.226–3.690	0.875	0.175	2.239	**0** **.027**	1.041
Sex (male, ref: female)	2.170	−3.936–8.275	3.083	0.054	0.704	0.483	1.017
OCD (dummy)	−23.556	−30.899–−16.214	3.708	−0.576	−6.354	**<0.001**	1.407
SIBL (dummy)	−11.768	−19.612–−3.924	3.961	−0.266	−2.971	**0** **.004**	1.377

Note: Dependent variable: Antisaccade Correct Response Rate (%), reference group: HC, B: Unstandardized regression coefficient, β: Standardized regression coefficient, CI: Confidence Interval, SE: Standard error, VIF: Variance Inflation Factor. Model statistics: Model I: *R*^2^ = 0.080, Adjusted *R*^2^ = 0.065, *F*_(2119)_ = 5.193, *p* = 0.007. Model II: *R*^2^ = 0.265, Adjusted *R*^2^ = 0.247, Δ*R*^2^ = 0.185, *F*_(3118)_ = 14.202, *p* < 0.001. Model III: *R*^2^ = 0.317, Adjusted *R*^2^ = 0.293, Δ*R*^2^ = 0.052, *F*_(4117)_ = 13.565, *p* < 0.001.

## Data Availability

The datasets generated and analyzed during the current study are available from the corresponding author upon reasonable request, subject to ethical and privacy restrictions. This study was not preregistered. The original experimental task materials are no longer available and therefore cannot be shared. No custom analysis code was generated for this study. Statistical analyses were performed using IBM SPSS Statistics (Version 27.0).

## Supplementary Materials

The following supporting information can be downloaded at: https://www.mdpi.com/article/10.3390/jemr19040078/s1, Table S1: Age-Adjusted Group Comparisons of Antisaccade Performance; Table S2: Comparison of Antisaccade Performance in the OCD Group by Medication Status; Table S3: Hierarchical Regression Analysis for Factors Predicting Antisaccade Latency; Table S4: Hierarchical Regression Analysis for Factors Predicting Antisaccade Velocity; Table S5: Sensitivity analyses using family-clustered robust standard errors for the final regression models predicting antisaccade performance.
