# Supplementary material for: Antisaccade Performance in Adolescent Obsessive–Compulsive Disorder: Evidence from a Familial-Risk Design
Source: J Eye Mov Res. 2026 Jul 15;19(4):78. doi: 10.3390/jemr19040078 (PMC13397853; doi:10.3390/jemr19040078)
Supplement: Supplementary file 1 [file jemr-19-00078-s001.zip › jemr-4403097-Supplementary Material.pdf]

Supplement Materials

Table S1. Age-Adjusted Group Comparisons of Antisaccade Performance

	OCD (*n*=48), AdjM(SE)	SIBL (*n*=35), AdjM(SE)	HC (*n*=39), AdjM(SE)	$F_{(2,116)}$	*p*	Partial η^2	Post-Hoc (Bonferroni)
Antisaccade Correct Response Rate (%)	42.43(2.48)	54.64 (2.86)	65.75 (2.80)	19.610	**< 0.001**	0.253	OCD-SIBL**(*p* = 0.005)**<br>OCD-HC **(*p* < 0.001)**<br>SIBL-HC **(*p* = 0.016)**
Mean Correct Antisaccade Latency (log-transformed)	−1.09 (0.05)	−1.25 (0.05)	−1.40 (0.05)	10.300	**< 0.001**	0.149	OCD–HC **(*p* < 0.001)**<br>OCD-SIBL(*p* = 0.082)<br>SIBL-HC (*p* = 0.111)
Mean Correct Antisaccade Velocity (log-transformed)	−1.31 (0.03)	−1.31 (0.04)	−1.21 (0.03)	2.984	0.054	0.048	NS

Notes: AdjM = age-adjusted estimated marginal mean derived from the ANCOVA model; therefore, these values differ slightly from the observed means presented in Table 2. SE = standard error. Age was entered as a covariate in all ANCOVA models. Latency and velocity analyses were performed using log-transformed values.

Table S2. Comparison of Antisaccade Performance in the OCD Group by Medication Status

Antisaccade Parameters	Medicated (n=27), med (IQR)	Unmedicated (n=21), med (IQR)	*Z*	*p*	*r*
**Antisaccade Correct Response Rate** (%)	39.47 (21.25–51.28)	46.80 (34.47–56.83)	1.361	0.173	0.196
Mean Correct Antisaccade Latency (s)	0.353 (0.298–0.401)	0.320 (0.291–0.336)	1.569	0.117	0.226
Mean Correct Antisaccade Velocity (°/ms)	0.254 (0.221–0.291)	0.274 (0.238–0.302)	1.444	0.149	0.208

med: Median, IQR: Interquartile range (Q1-Q3), *Z*: Mann-Whitney U, *r*: Rank Biserial Correlation

Table S3. Hierarchical Regression Analysis for Factors Predicting Antisaccade Latency

Variables	*B*	95% CI for *B*	*SE*	*β*	*t*	*p*	VIF
**Model I**							
Age	-0.023	-0.056–0.011	0.017	-0.121	-1.328	0.187	1.007
Sex (male, ref: female)	-0.043	-0.163–0.078	0.061	-0.064	-0.705	0.482	1.007
**Model II**							
Age	-0.015	-0.047–0.018	0.016	-0.078	-0.899	0.370	1.024
Sex (male, ref: female)	-0.021	-0.135–0.093	0.058	-0.032	-0.365	0.715	1.017
OCD (dummy)	0.231	0.114–0.349	0.059	0.339	3.894	**< 0.001**	1.028
**Model III**							
Age	-0.010	-0.042–0.022	0.016	-0.054	-0.629	0.531	1.041
Sex (male, ref: female)	-0.023	-0.136–0.090	0.057	-0.034	-0.402	0.688	1.017
OCD (dummy)	0.306	0.170–0.442	0.068	0.448	4.470	**< 0.001**	1.407
SIBL (dummy)	0.154	0.009–0.299	0.073	0.209	2.107	**0.037**	1.377

Note: Dependent variable: Mean correct antisaccade latency (s), reference group: HC, *B*: Unstandardized regression coefficient, *β*: Standardized regression coefficient, CI: Confidence Interval, *SE*: Standard error, VIF: Variance Inflation Factor, Model statistics: Model I: $R^2 = 0.020$, Adjusted $R^2 = 0.004$, $F_{(2,119)} = 1.217$, $p = 0.300$. Model II: $R^2 = 0.132$, Adjusted $R^2 = 0.110$, $\Delta R^2 = 0.112$, $F_{(3,118)} = 5.961$, $p = 0.001$. Model III: $R^2 = 0.163$, Adjusted $R^2 = 0.135$, $\Delta R^2 = 0.032$, $F_{(4,117)} = 5.711$, $p < 0.001$.

Table S4. Hierarchical Regression Analysis for Factors Predicting Antisaccade Velocity

Variables	*B*	95% CI for *B*	*SE*	*β*	*t*	*p*	VIF
**Model I**							
Age	0.010	-0.012–0.031	0.011	0.079	0.870	0.386	1.007
Sex (male, ref: female)	0.063	-0.014–0.140	0.039	0.147	1.619	0.108	1.007
**Model II**							
Age	0.008	-0.014–0.030	0.011	0.065	0.712	0.478	1.024
Sex (male, ref: female)	0.059	-0.019–0.136	0.039	0.136	1.498	0.137	1.017
OCD (dummy)	-0.048	-0.128–0.031	0.040	-0.110	-1.200	0.232	1.028
**Model III**							
Age	0.005	-0.017–0.027	0.011	0.040	0.446	0.657	1.041
Sex (male, ref: female)	0.060	-0.017–0.136	0.039	0.139	1.550	0.124	1.017
OCD (dummy)	-0.098	-0.190–-0.006	0.046	-0.223	-2.111	**0.037**	1.407
SIBL (dummy)	-0.102	-0.201–-0.004	0.050	-0.215	-2.063	**0.041**	1.377

Note: Dependent variable: Mean correct antisaccade velocity (°/ms), reference group: HC, *B*: Unstandardized regression coefficient, *β*: Standardized regression coefficient, CI: Confidence Interval, *SE*: Standard error, VIF: Variance Inflation Factor. Model statistics: Model I: $R^2 = 0.030$, Adjusted $R^2 = 0.013$, $F_{(2,119)} = 1.820$, $p = 0.167$. Model II: $R^2 = 0.041$, Adjusted $R^2 = 0.017$, $\Delta R^2 = 0.017$, $F_{(3,118)} = 1.698$, $p = 0.171$. Model III: $R^2 = 0.075$, Adjusted $R^2 = 0.043$, $\Delta R^2 = 0.036$, $F_{(4,117)} = 2.372$, $p = 0.056$.

Table S5. Sensitivity analyses using family-clustered robust standard errors for the final regression models predicting antisaccade performance

**Panel A. Antisaccade Correct Response Rate (%)**				
Variables	*B*	Robust *SE*	*t*	*p*
Age	1.958	0.770	2.542	**0.012**
Sex (male, ref: female)	2.170	3.005	0.722	0.472
OCD (dummy)	-23.556	4.063	-5.798	**< 0.001**
SIBL (dummy)	-11.768	3.455	-3.406	**< 0.001**
**Panel B. Mean Correct Antisaccade Latency, log-transformed**				
Variables	*B*	Robust *SE*	*t*	*p*
Age	-0.010	0.016	-0.655	0.514
Sex (male, ref: female)	-0.023	0.054	-0.422	0.674
OCD (dummy)	0.306	0.074	4.151	**< 0.001**
SIBL (dummy)	0.154	0.077	2.010	**0.046**
**Panel C. Mean Correct Antisaccade Velocity, log-transformed**				
Variables	*B*	Robust *SE*	*t*	*p*
Age	0.004	0.012	-0.412	0.681
Sex (male, ref: female)	0.060	0.040	1.498	0.137
OCD (dummy)	-0.098	0.046	-2.119	**0.036**
SIBL (dummy)	-0.102	0.048	-2.138	**0.035**

Note: Sensitivity analyses were performed using family-clustered robust standard errors, with family ID specified as the clustering variable. HC served as the reference group. Latency and velocity models were fitted using log-transformed dependent variables. *B*: unstandardized regression coefficient; *SE*: standard error.
